# Experience of and factors associated with violence against sexual and gender minorities in nine African countries: a cross-sectional study

**DOI:** 10.1186/s12889-021-10314-w

**Published:** 2021-02-15

**Authors:** Alex Müller, Kristen Daskilewicz, Mc Lean Kabwe, Anna Mmolai-Chalmers, Chelsea Morroni, Nelson Muparamoto, Adamson S. Muula, Vincent Odira, Martin Zimba, the Southern and Eastern African Research Collective for Health (SEARCH)

**Affiliations:** 1 Gender Health and Justice Research Unit, University of Cape Town, Cape Town, South Africa; 2 Department of Medical Ethics and History of Medicine, Universitätsmedizin Göttinge, Göttingen, Germany; 3 The Lotus Identity, Lusaka, Zambia; 4 Lesbians, Gays and Bisexuals of Botswana, Gaborone, Botswana; 5 Liverpool School of Tropical Medicine and Botswana UPenn Partnership, Gaborone, Botswana; 6 Department of Sociology, University of Zimbabwe, Harare, Zimbabwe; 7 College of Medicine, University of Malawi, Blantyre, Malawi; 8 Maaygo, Kisumu, Kenya; 9 Friends of Rainka, Lusaka, Zambia

**Keywords:** Violence, Sexual and gender minority, Sexual orientation and gender identity and expression, Africa, Discrimination, Lesbian, Gay, Bisexual, Transgender, Intersex

## Abstract

**Objective:**

The objective of this research was to assess physical and sexual violence experienced by sexual and gender minorities in nine African countries, and to examine factors associated with violence.

**Methods:**

We conducted an exploratory multi-country cross-sectional study among self-identifying sexual and gender minorities, using a survey tool available in paper and online. Participants were sampled through venue-based and web-based convenience sampling. We analysed data using descriptive statistics and logistic regression, with Stata15.

**Findings:**

Of 3798 participants, 23% were gender minorities, 20% were living with HIV, and 18% had been coerced into marriage. Fifty-six per cent of all participants had experienced physical or sexual violence in their lifetime, and 29% in the past year. Gender minorities had experienced significantly higher levels of violence compared to cisgender (sexual minority) participants. The variable most strongly associated with having experienced violence was being coerced into marriage (AOR, 3.02), followed by people living nearby knowing about one’s sexual orientation and/or gender identity (AOR, 1.90) and living with HIV (AOR, 1.47).

**Conclusion:**

Sexual and gender minorities in Eastern and Southern Africa experience high levels of violence. Sexual orientation and gender identity need to be recognised as risk factors for violence in national and regional law and policy frameworks. States should follow the African Commission Resolution 275 and provide protection against violence based on real or perceived sexual orientation or gender identity.

## Background

Violence against sexual and gender minorities is increasingly recognised as a key public health and human rights issue [1–5]. The United Nations (UN) Human Rights Council passed several resolutions to express concern about violence and discrimination against individuals motivated by their perceived or known sexual orientation and/or gender identity [6–9]. In 2014, the African Commission for Human and People’s Rights (ACHPR) passed Resolution 275, which calls on African states to offer protection from violence based on real or perceived sexual orientation and gender identity, and outlines specific obligations to achieve this [10]. In 2016, the UN Human Rights Council appointed the first *UN Independent Expert on Violence based on Sexual Orientation and Gender Identity* [11], who, in a recent report ([12]:1), noted that:

“[Violence and discrimination against sexual and gender minorities] are committed in all corners of the world, and victims are presumed to be in the millions, every year. These acts extend from daily exclusion and discrimination to the most heinous acts, including torture and arbitrary killings. At their root lie the intent to punish the non-conformity of victims with preconceived notions of what should be their sexual orientation or gender identity.”

Systematic reviews have demonstrated that sexual and gender minorities are more likely to be survivors of physical and sexual violence than the general population [2, 3]. A recent systematic review showed that the prevalence of violence against sexual and gender minorities that is motivated by bias against their sexual orientation and/or gender identity is also high [4]. However, the empirical studies included in these systematic reviews included little to no data from the African continent.

A joint dialogue of the African Commission for Human and People’s Rights, the Inter-American Commission on Human Rights and the UN highlighted this data gap and concluded that: “[d] ata and evidence is critical to understand the extent and gravity of violations and to advocate for the adoption of measures to prevent, address and redress human rights violations faced by [sexual and gender minorities]” ([13]:19). In his most recent report, the UN Independent Expert highlighted the lack of empirical data on violence against sexual and gender minorities in contexts outside of the United States and Europe as one of the biggest challenges [12].

Our exploratory study aimed to provide data on the experience of, and factors associated with, violence against sexual and gender minorities in nine African countries.

## Methods

### Study design and setting

We conducted an exploratory cross-sectional study in nine countries: Botswana, eSwatini, Ethiopia, Kenya, Lesotho, Malawi, South Africa, Zambia and Zimbabwe. With the exception of Lesotho and South Africa (and Botswana since June 2019), these countries retain colonial-era laws that criminalise same-sex sexuality, although the implementation of these laws varies greatly from country to country [14]. Following the guidelines for research with sexual and gender minorities in rights-constrained environments [15], we worked as a partnership between an academic research team and local community-based organisations, all of whom were equal decision-making partners. We carefully considered the security risks, challenges and options for sampling sexual and gender minority populations [16, 17] and recruited participants through venue-based and online methods, using convenience sampling.

### Participants, eligibility and data collection

Participants were eligible if they self-identified as a sexual and/or gender minority, were over the age of 18 and lived in one of the nine study countries. Data were collected with a phased approach, country per country, starting in countries with least risk (i.e., South Africa, eSwatini, Kenya), and ending in countries where data collection might pose the largest risks for data collectors and study participants (i.e., Ethiopia, Zambia). In each country, data were collected for 3 months. Overall, data collection took place between November 2016 and January 2019.

Due to safety and security concerns, we could not keep records of how many participants were approached. Overall, 4155 surveys were answered. Of those, 3798 surveys were included in the analysis. We excluded 357 surveys from our analysis: 67 due to protocol violations, and 290 due to incompleteness. These incomplete surveys only had data on participant characteristics, but no data on any of the outcomes (violence, mental health or health service use, see below). The participant characteristics of the excluded surveys showed no significant differences from the participant characteristics of included surveys.

### Measures

The survey was available in the nine languages most widely spoken in the study countries (Amharic, Chichewa, English, isiNdebele, Sesotho, Setswana, Shona, Siswati, Swahili). A hard copy of the survey was used for community-based data collection. Seventy-five per cent of surveys (*n* = 2862) were filled out in hard copy, and 25% online (*n* = 936). Of the hard copy surveys, 76% (*n* = 2170) were self-administered, and 24% (*n* = 692) were filled out with the help of a fieldworker in cases where participants requested assistance. An online version of the survey was hosted on REDCap (Vanderbilt University, Nashville), and the link was distributed through community organisations and individuals. The survey itself and more information about its components are available in our previously published research report [18].

In consultation with our community partner organisations, we created composite categorical variables for both sexual orientation and gender identity (SOGI). We asked each participant about sexual attraction (answer options, tick all that apply: women/ men/ transwomen/ transmen/ gender non-conforming people/ intersex people/ I do not feel sexual attraction/ other, please specify), sexual partners over their lifetime (answer options, tick all that apply: women/ men/ transwomen/ transmen/ gender non-conforming people/ intersex people/ I have not had sexual experiences/ other, please specify) and sexual identity (answer options: lesbian/ bisexual/ gay/ heterosexual/ asexual/ other, please specify). We further asked about sex assigned at birth and self-identified gender identity [19]: both were taken into account to group participants into gender identity categories. The sexual orientation variable takes into account sexual attraction, sexual behaviour, sexual identity and gender identity: we grouped participants into sexual orientation categories based on the best-fitting answer combination to these questions, giving preference to self-identification [16]. Participants whose answer combinations did not allow us to group them into the categories of lesbian, gay or bisexual [20] were grouped in the sexual orientation category ‘non-normative’. This included, for example, participants who identified as gender non-conforming and queer – without binary gender, conventional sexual orientation categories such as lesbian, gay or bisexual cannot be determined.

Our questions about socio-economic circumstances included age, religion, education, housing, employment and financial security. Additionally, we asked who knew about participants’ SOGI, which we recorded in binary variables (yes/no) for different groups of people (family members, friends, people in household, people living nearby, work colleagues).

To assess violence, we asked a series of yes/no questions about experiences and perpetrators of physical and sexual violence, both for lifetime and the past year. Additionally, we asked participants if they had been coerced into marriage. For participants who had experienced violence, we asked about three signs of post-traumatic stress disorder (PTSD): (1) flashbacks, nightmares or reliving of the traumatic incident, (2) avoidance behaviour and (3) feelings of jumpiness/ irritability/ restlessness. These were based on the diagnostic criteria for PTSD in the DSM-V [21]. We recoded participants who reported all three signs as ‘showing signs of PTSD’ in a binary variable.

A separate section of the survey asked about health service use and specific mental health outcomes. We do not report on these findings here but do include a binary variable about use of HIV-related healthcare as a proxy for living with HIV, which we hypothesised could be associated with violence because HIV infection is both a consequence of, and risk factor for, violence [22].

### Data analysis

All data were analysed with Stata15 (StataCorp, College Station).

For sample characteristics, we describe median and interquartile range (IQR) for continuous variables and percentages for categorical variables. For categorical outcome variables we describe proportions with 95% confidence intervals. We assessed bivariate and multivariate associations between experience of violence as the main outcome of interest and other variables. The outcome variable was defined in two ways: 1) lifetime experience of any form of violence (physical and/or sexual) and 2) past year experience of any form of violence (physical and/or sexual).

For analysis of bivariate associations with ‘experience of violence’, we considered the following variables based on a review of the literature (see, for example, [23–26]) and discussions with our community partner organisations: gender minority status (transgender women, transgender women or gender non-conforming participants), HIV status, rural location, coercion into marriage, financial and housing stability (living in formal versus informal housing), public knowledge of SOGI (among people living in the same household, and people living nearby). Variables that were significantly associated (*p* < 0.05) with the experience of violence outcome variables in bivariate analysis were included in the multivariate logistic regression models.

In the logistic regression models, associations with experience of violence were considered to be statistically significant if *p* < 0.05. We present odds ratios, *p*-values and we used 95% confidence intervals.

For missing data in descriptive statistics and bivariate analysis we describe levels of missingness. In the multivariate logistic regression models we used multiple imputation (MI) to minimise bias [27]. In our MI model, we assumed data to be missing at random, used chained equations and imputed categorical variables with predictive mean matching and binary variables with logistic regression. In the 290 surveys that were excluded due to incompleteness, participants had not provided information beyond initial participant characteristics. Therefore, these surveys were not included in the MI models.

### Regulatory compliance

The overall study was approved by the Human Research Ethics Committee of the University of Cape Town’s Faculty of Health Sciences (HREC 012/2016). Based on this approval, we gained approval from national ethics or health regulatory bodies. Table 1 lists the regulatory approvals in the specific study countries. We followed guidelines and established best practices for research on sexual and gender minorities’ health in rights-constrained environments [15, 28]: in some countries, obtaining regulatory approval would have significantly increased safety risks for our community partner organisations or research participants. Here, we initiated a community review process instead, where a review board of community members evaluated the risks and benefits of the study. The composition and review process of each community review board, as well as the evaluation of the study proposal, was overseen and approved by the Human Research Ethics Committee at the University of Cape Town’s Faculty of Health Sciences. Prior to enrolment, all participants provided informed consent. We ensured complete participant anonymity throughout the data collection process.

**Table 1:** Ethical and regulatory approval

Country	Approval authority and reference number
Botswana	Ethics Unit, Office of Research and Development, University of Botswana (UBR/RES/IRB/BIO/009) Health Research and Development Division, Ministry of Health and Wellness, Republic of Botswana (HPDME: 13/18/1)
eSwatini	Scientific and Ethics Committee, Ministry of Health and Social Welfare, Kingdom of Swaziland (no ref. number)
Ethiopia	Community review board
Kenya	Kenya Medical Research Institute (KEMRI/RES/7/3/1)
Lesotho	Research and Ethics Committee, Ministry of Health, Lesotho (ID94–2017)
Malawi	University of Malawi, College of Medicine Research and Ethics Committee (P.01/18/2330)
South Africa	University of Cape Town Faculty of Health Sciences Human Ethics Research Committee (HREC 012/2016)
Zambia	Community review board
Zimbabwe	Medical Research Council of Zimbabwe (MRCZ/A/2303)

## Results

### Characteristics of sample

The 3798 participants were diverse in their sexual orientations, and about one quarter (*n* = 887) of participants identified as a gender minority (transgender woman, transgender man or gender non-conforming; Table 2). The median age was 26 years. Only 42% of participants reported sufficient financial capacity to meet their everyday needs. Twenty per cent of participants were living with HIV, and 18% had been coerced into marriage.

**Table 2:** Characteristics of sample

Sample characteristics (*N* = 3798)		
**Age**		
Median (IQR)	26	18–59
Range		18–64
Missing (n, %)	180	4.74
**Country**	**n**	**%**
Botswana	618	16.27
eSwatini	104	2.74
Ethiopia	198	5.21
Kenya	976	25.70
Lesotho	173	4.56
Malawi	197	5.19
South Africa	832	21.91
Zambia	353	9.29
Zimbabwe	347	9.14
**Sexual orientation**	**n**	**%**
Lesbian	907	23.88
Bisexual	734	19.33
Bisexual women	202	5.32
Bisexual men	487	12.82
Gay	1686	44.39
Non-normative	270	7.11
Heterosexual^a^	185	4.87
Missing	16	0.42
**Gender identity**	**n**	**%**
Cisgender woman^a^	911	23.99
Cisgender man^a^	1911	50.32
Transgender woman	383	10.08
Transgender man	284	7.48
Gender non-conforming	188	4.95
Non-normative	32	0.84
Missing	89	2.34
**Characteristics**	**n**	**%**
Not enough money for basic needs (*N* = 3710)^b^	2131	56.11
Living in informal housing or on the street (*N* = 3780)	235	6.19
Living in rural area (*N* = 3763)	333	8.77
Accessing HIV care and treatment (*N* = 3651)	771	20.30
Coerced into marriage (*N* = 3570)	673	17.72
Living with people who know their SOGI	1256	33.07
Living near people who know their SOGI	899	23.67

*IQR* Interquartile Range, *SOGI* sexual orientation and/or gender identity

^a^Because the inclusion criteria were being a sexual minority and/or a gender minority, participants who identified as heterosexual are gender minorities (transgender women or men, or gender non-conforming people), and participants who identified as cisgender are sexual minorities (identified as lesbian, bisexual or gay)

^b^N varies due to missing data

### Experience of violence

Table 3 presents the proportion of participants who experienced physical and/or sexual violence, in their lifetime and the past year, and stratifies findings by sexual orientation, gender identity, age, HIV status and forced marriage. Overall, 56% of participants had experienced some form of violence in their lifetime. In the past year, 29% of participants had experienced some form of violence; 25% had experienced physical violence, and 19% sexual violence.

**Table 3:** Experience of violence in lifetime and past year among sexual and gender minority sample

	Any form of violence	Physical violence	Sexual violence
**Overall sample**	**% (95% CI)**	**% (95% CI)**	**% (95% CI)**
Lifetime	56.0 (54.4–57.7)	47.3 (45.7–49.0)	38.9 (37.3–40.5)
Past year	29.2 (27.8–30.7)	25.2 (23.8–26.6)	19.2 (17.9–20.5)
*By country (lifetime)*			
Botswana	38.7 (34.8–42.6)	31.1 (27.5–34.9)	26.5 (23.1–30.1)
Eswatini	72.8 (63.4–80.6)	58.3 (48.5–67.4)	49.5 (39.9–59.1)
Ethiopia	43.8 (36.2–51.6)	36.9 (29.7–44.6)	30.0 (23.4–37.6)
Lesotho	48.5 (41.0–56.1)	46.4 (38.9–54.0)	22.6 (16.9–29.6)
Kenya	61.3 (58.2–64.4)	53.0 (49.9–56.2)	43.9 (40.8–47.1)
Malawi	53.1 (46.0–60.0)	41.3 (34.6–48.4)	41.5 (34.8–49.0)
South Africa	65.3 (61.9–68.7)	55.0 (51.4–58.5)	47.9 (44.4–51.5)
Zambia	60.6 (55.1–65.8)	53.3 (47.8–58.8)	34.4 (29.4–40.0)
Zimbabwe	53.2 (47.8—58.6)	42.9 (37.7–48.4)	38.6 (33.5–44.0)
**By sexual orientation**	**% (95% CI)**	**% (95% CI)**	**% (95% CI)**
*Lesbian*			
Lifetime	56.8 (53.5–60.1)	47.6 (44.2–51.0)	39.7 (36.5–43.1)
Past year	28.1 (25.2–31.3)	24.4 (21.6–27.4)	16.1 (13.8–18.8)
*Bisexual (women)*			
Lifetime	62.0 (55.1–68.5)	54.5 (47.5–61.3)	47.5 (40.6–54.5)
Past year	32.7 (26.5–39.5)	27.0 (21.3–33.6)	20.1 (15.1–26.3)
*Bisexual (men)*			
Lifetime	47.2 (42.7–51.8)	40.1 (35.7–44.6)	31.5 (27.4–35.8)
Past year	25.4 (21.7–29.6)	22.2 (18.6–26.2)	17.5 (14.3–21.3)
*Gay*			
Lifetime	54.9 (52.5–57.4)	46.1 (43.7–48.6)	36.4 (34.1–38.8)
Past year	30.3 (24.7–31.4)	26.2 (24.1–28.4)	20.5 (18.6–22.5)
*Non-normative*			
Lifetime	68.1 (62.1–73.5)	58.8 (52.6–64.6)	54.1 (48.0–60.1)
Past year	28.4 (23.1–34.2)	23.5 (18.7–29.1)	20.8 (16.2–26.2)
**By gender identity**	**% (95% CI)**	**% (95% CI)**	**% (95% CI)**
*Transgender women*			
Lifetime	73.0 (68.2–77.3)	62.5 (57.4–67.3)	54.4 (49.2–59.4)
Past year	44.6 (39.5–49.8)	39.9 (35.0–45.0)	30.1 (25.6–35.0)
*Transgender men*			
Lifetime	54.4 (48.5–60.3)	46.3 (40.4–52.3)	40.6 (34.9–46.6)
Past year	32.8 (27.5–38.7)	29.1 (24.0–34.8)	24.2 (19.4–29.7)
*Gender non-conforming*			
Lifetime	69.8 (62.5–76.2)	59.9 (52.4–67.0)	56.4 (48.9–63.6)
Past year	36.8 (29.9–44.3)	31.2 (24.7–38.5)	24.6 (18.7–31.6)
**By age group**	**% (95% CI)**	**% (95% CI)**	**% (95% CI)**
*18–24 years*			
Lifetime	51.3 (48.7–54.0)	41.7 (39.1–44.3)	36.2 (33.7–38.7)
Past year	30.2 (27.9–32.7)	25.7 (23.5–28.1)	18.9 (16.9–21.0)
*25–34 years*			
Lifetime	58.7 (56.3–61.1)	51.3 (48.9–53.7)	40.4 (38.1–42.8)
Past year	29.5 (27.3–31.7)	25.7 (23.7–27.9)	20.0 (18.2–22.0)
*35–44 years*			
Lifetime	61.7 (56.3–66.8)	51.7 (46.3–57.0)	42.9 (37.6–48.3)
Past year	25.5 (21.0–30.5)	21.2 (17.1–26.0)	16.0 (12.4–20.4)
*45–54 years*			
Lifetime	60.5 (45.2–74.0)	51.2 (36.4–65.7)	37.2 (24.1–52.6)
Past year	9.3 (3.5–22.5)	9.3 (3.5–22.5)	2.3 (3.1–15.1)
*> 55 years*			
Lifetime	62.5 (26.5–88.5)	62.5 (26.5–88.5)	25.0 (5.7–64.9)
Past year	0 (no observations)	0 (no observations)	0 (no observations)
**By HIV status**			
*Living with HIV*			
Lifetime	65.9 (62.3–69.2)	57.0 (53.4–60.5)	46.8 (43.2–50.4)
Past year	40.2 (36.7–43.8)	35.4 (32.0–39.0)	26.8 (23.7–30.2)
*Not living with HIV*			
Lifetime	53.1 (51.2–55.0)	44.6 (42.7–46.5)	36.5 (34.7–38.3)
Past year	26.2 (24.6–27.9)	22.3 (20.8–23.9)	17.1 (15.7–18.5)
**By coerced marriage**			
*Forced to marry*			
Lifetime	75.6 (72.2–78.7)	67.3 (63.6–70.7)	56.2 (52.4–59.9)
Past year	46.8 (43.1–50.7)	41.5 (37.8–45.3)	33.8 (30.3–37.5)
*Not forced to marry*			
Lifetime	51.4 (49.5–53.2)	42.6 (40.8–44.4)	34.6 (32.9–36.4)
Past year	24.8 (23.2–26.4)	21.2 (19.7–22.7)	15.4 (14.1–16.7)

*CI* confidence interval

Experiences of violence were high across participants of all sexual orientations and gender identities. Transgender women had experienced the highest levels of violence: three in four transgender women (73%) had experienced any form of violence in their lifetime, and almost half (45%) in the past year. Compared to cisgender (sexual minority) participants, gender minority participants had experienced significantly higher levels of any form of violence in their lifetime (*p* < 0.01 for both; data not shown). Younger sexual and gender minority people experienced higher levels of recent violence, with 30% of 18–24 year olds having experienced some form of violence in the past year, compared to 25% of 35–44 year olds and 9% of participants over the age of 45.

Both lifetime and past year experiences of some form of violence were significantly higher among participants who were living with HIV, compared to participants not living with HIV (*p* < 0.01); and among participants who reported being coerced into marriage, compared to participants not coerced into marriage (*p* < 0.01; see also Table 3).

Of participants who had experienced violence in their lifetime, 70% (95% CI, 68.2–72.2) believed that it had been motivated by their sexual orientation and/or gender identity (SOGI), 44% (95% CI, 41.4–45.9) reported three signs of PTSD, and 25% (95% CI, 23.2–27.0) were living with HIV. Of participants who had experienced violence in the past year, 76% (95% CI, 74.8–80.1) believed it was SOGI-motivated, 46% (95% CI, 43.2–49.6) reported three signs of PTSD and 29% (95% CI, 26.4–32.0) were living with HIV (data not shown).

### Factors associated with experience of violence

In bivariate analysis, variables significantly associated with lifetime experiences of violence were: coercion into marriage, having people living nearby and people living in the same household know one’s SOGI, being a gender minority, HIV status, and living in informal housing (Table 4). The same variables were also significantly associated with experience of violence in the past year.

**Table 4:** Bivariate associations with lifetime and past year violence

	Lifetime experience of violence*		Past year experience of violence*	
*Variable*^a^	*OR* *(95% CI)*	*p-value*	*OR* *(95% CI)*	*p-value*
Coerced into marriage	2.93 (2.42–3.55)	< 0.01	2.68 (2.25–3.18)	< 0.01
People living nearby know SOGI	2.17 (1.84–2.55)	< 0.01	1.39 (1.18–1.64)	< 0.01
People living within same household know SOGI	1.77 (1.54–2.05)	< 0.01	1.24 (1.06–1.44)	< 0.01
Being a gender minority	1.72 (1.47–2.02)	< 0.01	1.76 (1.49–2.07)	< 0.01
Living with HIV	1.70 (1.44–2.02)	< 0.01	1.89 (1.59–2.24)	< 0.01
Living in informal housing	0.59 (0.44–0.78)	< 0.01	0.52 (0.40–0.69)	< 0.01
Survey taken online	1.12 (0.96–1.32)	0.16	0.62 (0.51–0.74)	< 0.01

*OR* Odd’s Ratio, *CI* Confidence Interval, *SOGI* sexual orientation and/or gender identity

*only reporting associations significant at *p* < 0.05

^a^N varies due to missing data; variable N for lifetime experience of violence: Coerced into marriage (*N* = 3555), People living nearby know SOGI (3590), People living within same household know SOGI (*N* = 3590), Being a gender minority (*N* = 3517), Living with HIV (*N* = 3464), Living in informal housing (*N* = 3585); variable N for past year experience of violence: Coerced into marriage (*N* = 3538), Living with HIV (*N* = 3450), Being a gender minority (*N* = 3498), People living nearby know SOGI (*N* = 3571), People living within same household know SOGI (*N* = 3571), Survey taken online (*N* = 3583), Living in informal housing (*N* = 3567)

Table 5 presents the variables that showed independent significant associations with lifetime and past year experience of violence in multivariate logistic regression analysis; these were similar for both outcomes. The variable most strongly associated with having experienced lifetime and past year violence was being coerced into marriage (lifetime adjusted odds ratio (AOR) 3.02; past year AOR 2.72). Other factors significantly and positively associated with these outcomes were people living nearby knowing about one’s sexual orientation and/or gender identity (lifetime AOR 1.90; past year AOR 1.42), and living with HIV (lifetime AOR 1.47; past year 1.63).

**Table 5:** Multivariate analysis for factors associated with lifetime and past year experience of violence

	Lifetime experience of violence*		Past year experience of violence*	
*Variable*	*OR* *(95% CI)*	*p-value*	*OR* *(95% CI)*	*p-value*
Coerced into marriage	3.02 (2.48–3.69)	< 0.01	2.72 (2.27–3.27)	< 0.01
Gender identity (reference category cisgender man)				
Transgender woman	2.51 (1.88–3.36)	< 0.01	2.19 (1.67–2.88)	< 0.01
Gender non-conforming	2.42 (1.64–3.58)	< 0.01	1.87 (1.29–2.71)	< 0.01
Cisgender woman	1.94 (1.48–2.55)	< 0.01	1.59 (1.14–2.23)	< 0.01
People living nearby know SOGI	1.90 (1.56–2.31)	< 0.01	1.42 (1.16–1.74)	< 0.01
Living with HIV	1.47 (1.23–1.77)	< 0.01	1.63 (1.35–1.97)	< 0.01
Not enough money for everyday needs	0.82 (0.71–0.95)	< 0.01	0.63 (0.53–0.75)	< 0.01

*AOR* adjusted odd’s ratio, *CI* confidence interval, *SOGI* sexual orientation and/or gender identity

* only reporting associations significant at *p* < 0.05

Compared to cisgender (sexual minority) men, the likelihood of experiencing lifetime violence was higher for transgender women (AOR, 2.51; 95% CI, 1.88–3.36, *p* < 0.01), for gender non-conforming people (AOR, 2.42; 95% CI, 1.64–3.58, *p* < 0.01) and for cisgender (sexual minority) women (AOR, 1.94; 95% CI, 1.48–2.55, *p* < 0.01).

## Discussion

Our exploratory study provides the first large-scale data on sexual orientation, gender identity and violence from the African continent. It shows that sexual and gender minorities in Southern and Eastern Africa face high levels of violence, and that often, sexual and gender minorities perceive this violence to be motivated by their sexual orientation and/or gender identity. Whilst more than half of all sexual and gender minorities in our study had experienced violence, bisexual women, transgender women and gender non-conforming people seem to be most at risk.

Our study is subject to some important limitations. First, it is an exploratory study, and our study design was not robust enough to investigate causality or protective factors. As such, it cannot identify predictors or risk factors for violence, and only reports factors that are associated with violence. Second, there are no standardised ways of asking about SOGI [16], or violence, and this limits the comparability of our findings. Third, others have found that community venue-based sampling might overestimate health concerns [29]. We have tried to mitigate this by broadening our sample through sampling online [17]. It is important to note that the levels of experiences of violence did not significantly differ between community venue-based and online sample. Where we did see a significant difference, it seemed due to the difference between fieldworker and self-administered surveys, which is in line with observations in other studies [30], which also found higher levels of reported violence in self-administered surveys. Lastly, we found some associations that were opposite to what we expected (for example, informal housing was negatively associated with violence). These are likely confounded due to other unmeasured variables.

Despite these limitations, our findings are congruent with and confirm grey literature findings of high levels of violence experienced by sexual and gender minorities in the study countries (see, for example, [23–26, 31]), as well as findings of high levels of violence experienced by sexual and gender minority people worldwide [2–4]. Our findings highlight that especially younger sexual and gender minority people are experiencing high levels of violence.

Compared to the levels of violence experienced by the general population in each country (assumed to be cisgender and heterosexual), sexual and gender minority people in our study reported higher levels of violence. For example, in Botswana, studies suggest that 10–11% of women in the general population have experience sexual violence in their life [32, 33], compared to 27% of sexual and gender minority people in our study. The Kenyan Demographic and Health Survey (DHS) from 2014 estimates that 39% of women in the general population have experienced physical violence in their lifetime [34], compared to our findings of 53% of Kenyan sexual and gender minority people. The 2015 Zimbabwe DHS found that one in seven women in the general population (14%) had experienced sexual violence in their lifetime [35]. Among Zimbabwean sexual and gender minority people in our study, 39% had experienced sexual violence. While the comparability of these studies might be limited, the much higher levels of violence reported by sexual and gender minorities in our study seem to indicate reasons beyond methodological differences. This suggestion is confirmed by literature from other contexts, which has consistently found higher levels among sexual and gender minorities compared to the general population [2, 3].

Yet, at the same time, sexual and gender minorities are usually not catered for in violence prevention and survivor support services [36]. Worse, the laws that criminalise same-sex sexuality in six of the nine countries of our study often actively discourage sexual and gender minority survivors of violence to report experiences of violence to the police or to seek healthcare for fear of arrest, intimidation or blackmail [31, 37]. Besides the laws, harmful social attitudes and norms born out of patriarchal heterosexism do not only harm sexual and gender minority people directly (ie. through violence), but also indirectly by impeding access to  healthcare and justice. Studies from Southern Africa have shown that sexual orientation and gender identity-related prejudice leads to discrimination of sexual and gender minorities in healthcare and the criminal justice system, including when seeking care after experiencing violence [36, 38–40].

Our study found that having been coerced into (heterosexual) marriage held the strongest association with experiencing violence. We are not aware of other work that has looked at the relationship between coerced marriage and violence among sexual and gender minorities. We think that there might be a few explanations for this. One, sexual and gender minorities who are coerced into marriage could be more likely to live in communities that hold conservative, heteropatriarchal normative ideas about religion, gender roles, family and social structures – which could be the reason they are coerced into marriage in the first place. It is likely that homo- and transphobic attitudes are more prevalent in these communities, which would also increase the risk for experiencing SOGI-related violence. Conservative, heterosexist contexts are well documented in most of the countries of our research [41, 42], and other researchers have shown how violence has been used to police (and discourage) non-conforming sexual orientations and gender identities [23, 43]. Two, it is possible that sexual and gender minority people in coerced heterosexual marriages are at higher risk for violence by their spouse if their sexual orientation or gender identity is revealed, and being forced to have sex in coerced heterosexual marriages is a form of sexual violence itself. Three, it is also possible that sexual and gender minority people who have been the targets of violence feel that heterosexual marriage might offer them protection from SOGI-motivated violence, and consider this reason for marriage a form of coercion. Lastly, heterosexual marriage might provide economic and financial stability, which is especially important for sexual and gender minority people who are economically precarious, which is also confirmed by our findings. We recommend conducting qualitative research to better understand our quantitative findings.

## Conclusion

Our study provides important evidence on the high levels of violence experienced by sexual and gender minority people in Eastern and Southern Africa. Our findings highlight the importance of the African Commission’s Resolution 275 [10] and emphasise the need for states to protect sexual and gender minority people from violence, following the practical steps outlined in the Ekurhuleni Declaration [44]. States need to be willing to enforce their existing protective legal provisions (for example, the right to non-discrimination) equally to protect sexual and gender minorities. Further, states should repeal provisions that criminalise same-sex sexuality and/or non-conforming gender expression in order to reduce the vulnerability of sexual and gender minority people and increase their access to healthcare, justice and social support systems. Beyond legal reform, heteropatriarchal and heterosexist social and cultural norms that place sexual and gender minority people at risk for violence, including those that exist among healthcare providers, need to be challenged through on-going education by states and civil society organisations. An awareness of the severe and complex vulnerabilities to violence that sexual and gender minority people face is essential for efforts to reduce such violence.

## Data Availability

The datasets generated during the current study are not publicly available in order to minimise the risk of deliberate misinterpretation or misuse to further state-sponsored homo- and transphobia. The dataset includes data on mental ill-health. Given the long history of pathologisation of non-conforming sexual orientations and gender identities, the authors fear that data that negatively represents the negative mental health consequences of homo- and transphobia might be purposefully misinterpreted to further homo- and transphobic arguments. Data are available from the corresponding author upon reasonable request and with permission of the other partners of the *Southern and East African Research Collective for Health*.

## Abbreviations

PTSD: Post-traumatic stress disorder
SOGI: Sexual orientation and gender identity
